# Supplementary material for: Raspberry Ketone-Mediated Inhibition of Biofilm Formation in Salmonella enterica Typhimurium—An Assessment of the Mechanisms of Action
Source: Antibiotics (Basel). 2023 Jan 23;12(2):239. doi: 10.3390/antibiotics12020239 (PMC9952675; doi:10.3390/antibiotics12020239)
Supplement: Supplementary file 1 [file antibiotics-12-00239-s001.zip › Supplementary Data- Antibiotics-Research Article-23123.pdf]

Raspberry Ketone-Mediated Inhibition of Biofilm Formation in *Salmonella enterica* Typhimurium– An Assessment of the Mechanisms of Action

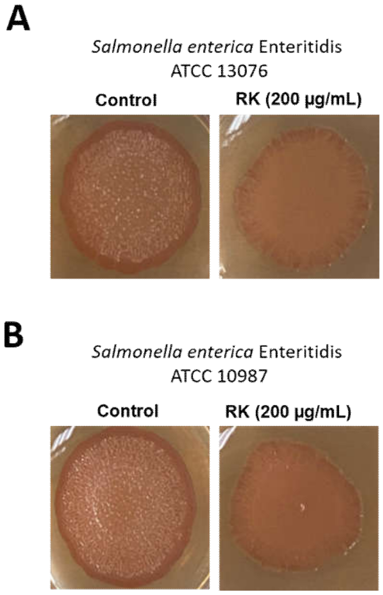

Figure S1. Biofilms of *S. enterica* Enteritidis ATCC 13076 and *S. enterica* Enteritidis SJTUF 10976 strains grown for 4 days on Congo red agar plates with or without RK at 28 ˚C.

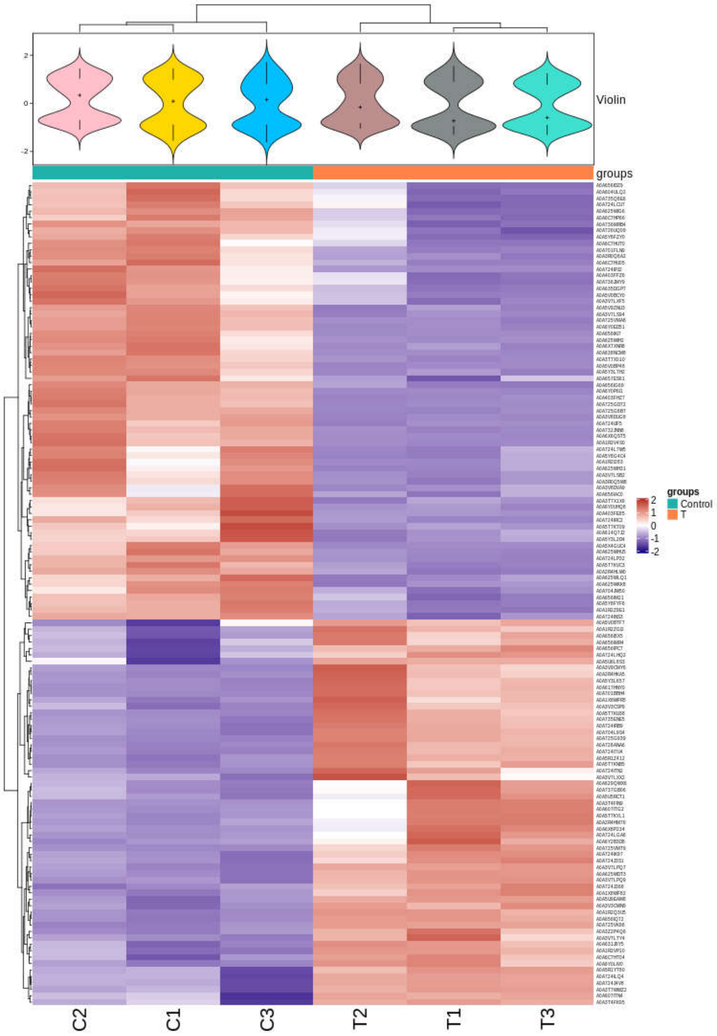

Figure S2. Protein profiles of *S.* Typhimurium biofilm with or without RK treatment. Hierarchical clustering of differential level proteins from three biological replicates of control and RK-treated *S.* Typhimurium biofilm. Red represents the highest relative expression, and blue represents the lowest. C1, C2, and C3 (*S.* Typhimurium controls); T1, T2, and T3 (RK treated *S.* Typhimurium).

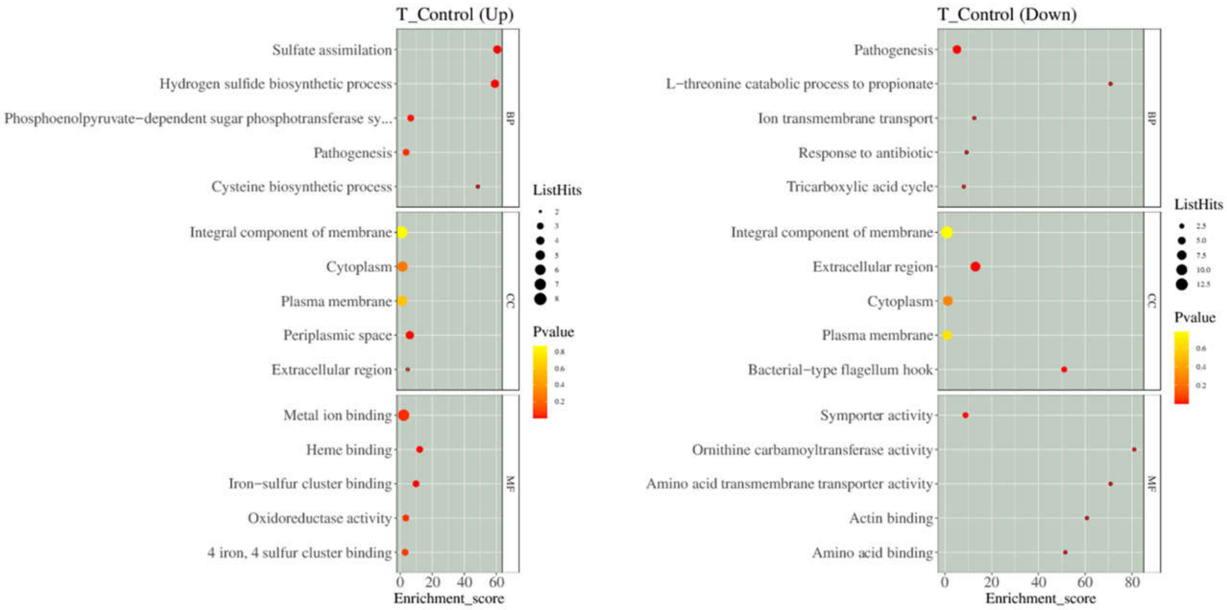

Figure S3. GO analysis of differentially expressed proteins in *S.* Typhimurium biofilm treated with RK.

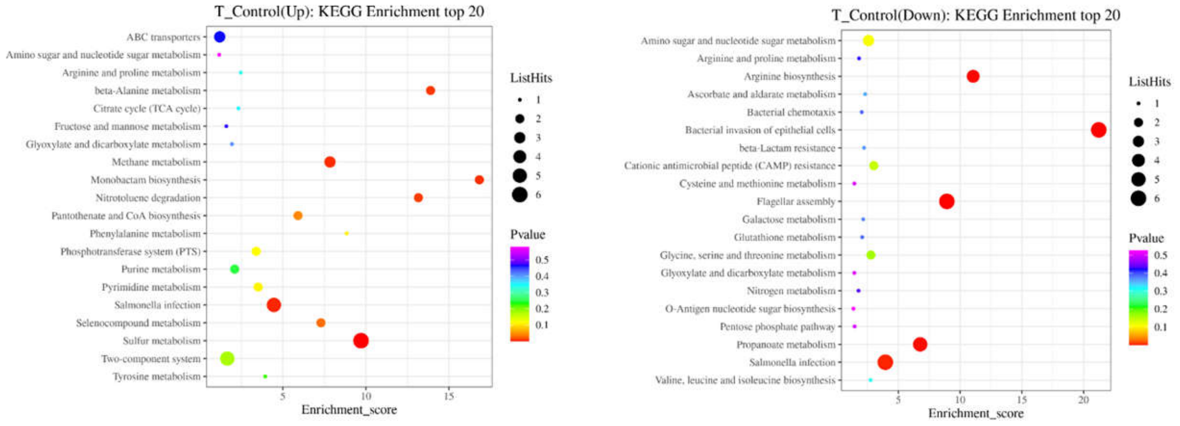

Figure S4. KEGG pathway enrichment of metabolic pathways involved in *S.* Typhimurium biofilm treated with RK.

Table S1. List of primers used

Gene	Primer Sequence
csgD	R: CCGCGTTATCCAGGTTGTTG<br>F: ACGCTACTGAAGACCAGGAAC
csg B	R: GCATTCGCCACGCAGAATA<br>F: CCAACGATGCCAGTATATCG<br>R: TGGCCTTATTTCCAGAACCT

Table S2. List of 128 differentially expressed proteins after RK treatment (fold change > 1.5 and $p < 0.05$)

Accession	Gene Name	Description	Fold Change
A0A725GD72	csgB	Curlin minor subunit CsgB	0.122
A0A732JNN8	G4G70_004163	Curli production assembly/transport component CsgF	0.244
A0A638NCM8	sipC	SPI-1 type III secretion system needle tip complex protein SipC	0.245
A0A3T7X010	arcA	Arginine deiminase	0.261
A0A5T7KVC3	R841_14175	Uncharacterized protein	0.275
A0A656IKI7	A673_01937	L-threonine dehydratase catabolic TdcB	0.285
A0A6Y0P6I1	csgD	Transcriptional regulator CsgD	0.287
A0A6Y0ED51	tdcC	Threonine/serine transporter TdcC	0.300
A0A656IG69	A673_02422	Putative arginine/agmatine antiporter	0.310
A0A724IPJ2	flgL	Flagellar hook-filament junction protein FlgL	0.315
A0A1R2V4S0	csgC	Curli assembly protein	0.335
A0A6X6QST5	flgK	Flagellar hook-associated protein 1	0.349
A0A403FHZ7	yccT	UPF0319 protein YccT	0.350
A0A725G8B7	csgG	Curli production assembly/transport component CsgG	0.353
A0A5X4GUC4	EGM21_03930	Porin OmpD	0.355
A0A3R0Q8A2	potE	Putrescine transporter PotE	0.368
A0A5V0BCY0	DUU06_22760	Uncharacterized protein	0.374
A0A625WKK8	ompF	Porin OmpF	0.404
A0A738NT28	waaO	Lipopolysaccharide 3-alpha-galactosyltransferase	0.419
A0A403FFZ6	argF	Ornithine carbamoyltransferase	0.420
A0A3V7LS94	sopE2	Guanine nucleotide exchange factor	0.424
A0A724LP32	G2712_17610	Effector protein SopB	0.428
A0A625WIH2	spaN	SPI-1 type III secretion system protein SpaN	0.434
A0A3V8DUG8	sipB	SPI-1 type III secretion system needle tip complex protein SipB	0.443
A0A3V7LSB2	fliD	Flagellar hook-associated protein 2	0.451
A0A6X7XNR8	speF	Ornithine decarboxylase SpeF	0.464
A0A701FLN9	tdcD	Propionate kinase	0.466
A0A724IJF5	G2712_06750	SPI-1 type III secretion system effector SipA	0.470
A0A635DGP7	arcC	Carbamate kinase	0.486
A0A6C7HU05	tdcA	TDC operon transcriptional activator	0.505
A0A656IDZ9	A673_04814	Transporter, major facilitator family protein	0.511
A0A704PHQ1	G0G55_23700	Uncharacterized protein	0.514
A0A6X6QWG1	GI501_03870	LPS O-antigen length regulator	0.517
A0A625WH31	AIU46_11590	2-methylcitrate dehydratase	0.529
A0A656IAC0	A673_04622	Uncharacterized protein	0.537
A0A604ULQ2	chiP	Chitoporin	0.542

A0A625WHU5	sptP	Protein-tyrosine-phosphatase	0.543
A0A5V0BP48	hcp	Type VI secretion system tube protein Hcp	0.551
A0A736JMY9	argF	Ornithine carbamoyltransferase	0.555
A0A736MRB4	GNA89_003780	Gate domain-containing protein	0.566
A0A403FE85	DTI44_07395	HAAAP family serine/threonine permease	0.566
A0A1R2U5W0	A3S27_03910	DUF1471 domain-containing protein	0.572
A0A1R2Z9E1	motA	Flagellar motor stator protein MotA	0.573
A0A3V7LXF5	gatY	D-tagatose-1,6-bisphosphate aldolase subunit GatY	0.580
A0A5V9ZNU3	AU760_19015	Dyp-type peroxidase	0.587
A0A656IFX7	A673_02423	Lysine decarboxylase, inducible	0.590
A0A5Y8FYF6	fliZ	Flagella biosynthesis regulatory protein FliZ	0.591
A0A2R4HLW0	tdcG	L-serine dehydratase	0.597
A0A724L7W5	G2712_04480	Citrate synthase	0.597
A0A5Y8FZY0	nanM	N-acetylneuraminate epimerase	0.601
A0A6C7HUT0	tdcE	Formate C-acetyltransferase	0.601
A0A3R0Q5W8	DTI44_11695	Uncharacterized protein	0.601
A0A5Y3L7H2	cbiD	Cobalt-precorrin-5B C(1)-methyltransferase	0.605
A0A724LCU7	G2712_17825	Gfo/Idh/MocA family oxidoreductase	0.607
A0A6Y0UHQ8	GJG76_22260	TolC family protein	0.608
A0A614Q7J2	sstT	Serine/threonine transporter SstT	0.610
A0A3V8DVA9	EZV86_03500	Uncharacterized protein	0.611
A0A6C7HVE6	sicA	Type III secretion system translocator chaperone SicA	0.612
A0A725PL70	sopD	SPI-1 type III secretion system effector SopD	0.616
A0A1R2I283	yciF	DUF892 family protein	0.617
A0A704JW50	G0G38_23300	Class I SAM-dependent methyltransferase	0.619
A0A5Y8G4C4	prpB	2-methylisocitrate lyase	0.619
A0A724IRC2	G2712_12205	Ig-like domain repeat protein	0.620
A0A6C7HP66	fliL	Flagellar protein FliL	0.622
A0A3T7X1X6	DUU06_21425	MotA_ExbB domain-containing protein	0.626
A0A725VMA8	cptA	Phosphoethanolamine transferase CptA	0.631
A0A726UQ09	G2939_19075	UDP-glucose 6-dehydrogenase	0.631
A0A625WIG6	arnA	Bifunctional polymyxin resistance protein ArnA	0.637
A0A6Y2B3D8	GBX02_08520	TolC family outer membrane protein	1.604
A0A607ITN4	AIU46_22405	Sulfate ABC transporter substrate-binding protein	1.623
A0A656IQ72	A673_00334	Isochorismatase family protein	1.635
A0A725VMT9	G2853_21420	Type I secretion system permease/ATPase	1.635
A0A1X8WF82	ABA47_0250	SnoaL-like domain	1.642
A0A635DBH6	yicI	Alpha-xylosidase	1.660
A0A656IMR4	A673_01060	Uncharacterized protein	1.664
A0A656IPC7	A673_00726	Uncharacterized protein	1.664
A0A3V3CSP9	AIU46_05390	DUF2756 family protein	1.687

A0A5U9EAM8	yciU	UPF0263 protein YciU	1.68
A0A724ILQ4	cysJ	Sulfite reductase [NADPH] flavoprotein alpha-component	1.692
A0A724ITN2	G2712_19065	SPI-2 type III secretion system effector SopD2	1.700
A0A1X8WFR5	A3S27_02145	Uncharacterized protein	1.707
A0A5T7KNB5	acnA	Aconitate hydratase	1.712
A0A3T4FK95	DUU06_11840	Sulfate ABC transporter substrate-binding protein	1.729
A0A724J368	G2712_01610	SPI-2 type III secretion system chaperone SseA	1.735
A0A605GP63	hdeB	Acid stress chaperone HdeB	1.758
A0A2R4HKA5	dsbE	Cytochrome c biogenesis protein CcmG	1.770
A0A656IF23	A673_02740	PTS system sorbose subfamily IIB component	1.773
A0A631J8Y5	B7900_07485	Uncharacterized protein	1.782
A0A1R2VP10	A3S27_13145	DUF2501 domain-containing protein	1.819
A0A2R4HM76	A3S27_11625	Anaerobic sulfatase maturase	1.831
A0A3V3CWN9	psiF	Phosphate starvation-inducible protein PsiF	1.846
A0A724J3S1	G2712_03435	SIS domain-containing protein	1.859
A0A724IRB9	G2712_08240	NAD-dependent dihydropyrimidine dehydrogenase subunit PreT	1.868
A0A3Z2P4Q6	A4J95_24425	Uncharacterized protein	1.873
A0A3V9CMY6	ccmE	Cytochrome c-type biogenesis protein CcmE	1.884
A0A3V7LTY4	AIU46_12710	Isochorismatase family protein	1.884
A0A3E1XB95	CRE05_18705	Uncharacterized protein	1.885
A0A617HNY0	AQ518_03845	Hydrogenase 1 large subunit	1.902
A0A5U5RCT1	spvD	SPI-2 type III secretion system effector cysteine hydrolase SpvD	1.912
A0A625WDT3	AIU46_06235	PTS system mannose/fructose/N-acetylgalactosamine-transporter subunit IIB	1.923
A0A3T7WWZ2	cysN	Sulfate adenylyltransferase subunit 1	1.949
A0A5T7KYL1	R841_17330	Arylsulfatase	1.965
A0A656I8X5	A673_04830	Uncharacterized protein	1.970
A0A725VK96	G2816_11195	Methyl viologen efflux MFS transporter SmvA	1.994
A0A724I7U4	G2712_13365	SPI-2 type III secretion system effector deubiquitinase SseL	2.030
A0A5T7KU38	R841_10450	Cytochrome c-type biogenesis protein	2.031
A0A704L9S4	preA	Dihydrothymine dehydrogenase	2.126
A0A737GB06	spvB	Mono(ADP-ribosyl)transferase SpvB (Fragment)	2.128
A0A656IN85	A673_01662	Porin OmpL	2.169
A0A724LGA6	G2712_08075	APC family permease	2.174
A0A3V7LPQ9	AIU46_06245	PTS system mannose/fructose/sorbose family transporter subunit IID	2.244

A0A724N7K8	G2427_03735	Histidine kinase	2.244
A0A735ENE5	G4K89_003466	Linear amide C-N hydrolase	2.305
A0A5R1Z412	C9F08_02585	DUF1471 domain-containing protein	2.311
A0A3T4FIN9	DUU06_12575	FAD-binding oxidoreductase	2.352
A0A703RUJ1	G0F99_18590	Porin	2.358
A0A629QWX6	spvC	MAPK phosphothreonine lyase	2.394
A0A625AX66	AIE92_08150	DUF1471 domain-containing protein	2.437
A0A724IK97	G2712_03440	SIS domain-containing protein	2.440
A0A626EIC9	A3U78_20300	DotA/TraY family protein	2.467
A0A5Y3L657	CBS77_06260	NiFe hydrogenase	2.476
A0A6X8P234	GB400_04910	DUF861 domain-containing protein	2.484
A0A607ITG2	AIU46_10125	NAD-dependent phenylacetaldehyde dehydrogenase	2.640
A0A724IJI3	asr	Acid shock protein	2.680
A0A701BBH4	torD	Chaperone protein TorD	3.115
A0A725G939	torA	Trimethylamine-N-oxide reductase	3.257
A0A3V7LPQ7	AIU46_06240	PTS mannose/fructose/sorbose/N-acetylgalactosamine transporter subunit IIC	3.282
A0A726ANA6	torC	Cytochrome c-type protein	3.838
